# Ionization injection of highly-charged copper ions for laser driven acceleration from ultra-thin foils

**DOI:** 10.1038/s41598-018-37085-6

**Published:** 2019-01-24

**Authors:** Jun Li, Alexey V. Arefiev, Stepan S. Bulanov, Daiki Kawahito, Mathieu Bailly-Grandvaux, George M. Petrov, Christopher McGuffey, Farhat N. Beg

**Affiliations:** 1 Center for Energy Research, University of California San Diego, La Jolla, CA 92093 USA; 2 Department of Mechanical and Aerospace Engineering, University of California San Diego, La Jolla, CA 92093 USA; 3 Lawrence Berkeley National Laboratory, Berkeley, CA 94720 USA; 4 Naval Research Laboratory, Plasma Physics Division, Washington, DC 20375 USA

## Abstract

Laser-driven ion acceleration is often analyzed assuming that ionization reaches a steady state early in the interaction of the laser pulse with the target. This assumption breaks down for materials of high atomic number for which the ionization occurs concurrently with the acceleration process. Using particle-in-cell simulations, we have examined acceleration and simultaneous field ionization of copper ions in ultra-thin targets (20–150 nm thick) irradiated by a laser pulse with intensity 1 × 10^21^ W/cm^2^. At this intensity, the laser pulse drives strong electric fields at the rear side of the target that can ionize Cu to charge states with valence L-shell or full K-shell. The highly-charged ions are produced only in a very localized region due to a significant gap between the M- and L-shells’ ionization potentials and can be accelerated by strong, forward-directed sections of the field. Such an “ionization injection” leads to well-pronounced bunches of energetic, highly-charged ions. We also find that for the thinnest target (20 nm) a push by the laser further increases the ion energy gain. Thus, the field ionization, concurrent with the acceleration, offers a promising mechanism for the production of energetic, high-charge ion bunches.

## Introduction

Ion acceleration by a high intensity (>10^18^ W/cm^2^) laser pulse interacting with a solid target has been an active research field in the past two decades^1–3^. The major interest stems from the prospects of designing and building a novel laser-driven ion source that is significantly more compact in size and shorter in duration than conventional ion accelerators. The potential applications of laser-driven ion beams have been proposed and studied in both science and technology fields, including cancer therapy^4–6^, isochoric heating^7–9^, proton radiography^10,11^, and ion-driven fast ignition^12–14^. Ions heavier than protons can have advantages in cancer therapy^15^ and can also be beneficial for other applications, such as generation of nuclear reactions^16,17^, heavy element synthesis^18^, and production of exotic isomers and isotopes for biomedical use. Numerous ion acceleration mechanisms that involve intense laser-solid interactions have already been identified. These include target normal sheath acceleration (TNSA)^19–22^, radiation pressure acceleration (RPA)^23–26^, shock wave acceleration (SWA)^27,28^, acceleration based on relativistic induced transparency (RIT)^29–31^, and magnetic vortex acceleration (MVA)^32–35^.

Despite the improved understanding of the underlying acceleration mechanisms, the generation of laser-driven ion beams with sufficient beam quality to attain broad use for different applications remains elusive, with the exception of proton radiography and deflectometry that are routinely used to probe target density and fields in laser-matter interaction experiments. Some of the mechanisms require a very high laser contrast in order to be realized experimentally at existing laser facilities and to deliver high ion energies needed by applications. One mechanism that is comparatively robust and easy to implement experimentally, TNSA (which is predominantly used for proton radiography), offers little control over the accelerated ion spectrum and is not very efficient, particularly for ions with higher atomic number than that of carbon. The resulting ion beams are typically divergent and have a broad, exponentially decreasing energy spectrum. Therefore, development of extra “control knobs” for the already practiced acceleration mechanisms^36^ would be highly advantageous, as this would increase their viability for numerous applications.

Here, we demonstrate that for Cu, an example period-four transition metal, the coincidence of field ionization with ion acceleration can provide an extra degree of control over the generated ion spectrum and enable generation of energetic bunches of ions with high charge state, Z. Laser-driven ion acceleration is often analyzed assuming that the target ionization reaches a steady state very early in the interaction with the laser pulse as if the ionization process were instantaneous. This may be a fair simplification for laser-interactions with carbon-based targets. However, for commonly-used metals, the validity of such an assumption is questionable. In the extreme of high atomic number materials such as Au or Pt, the ion acceleration does not occur before the peak of the laser pulse^37^.

**Figure 1:**
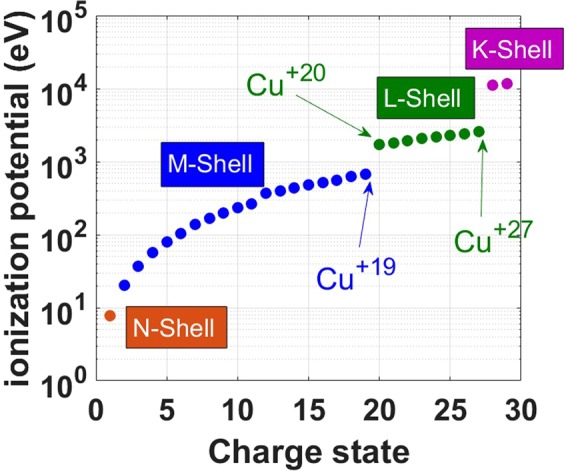
Ionization energy needed to reach charge state Z from charge state Z-1 of copper ions, where the color-coding indicates the valence electron shell of state Z-1.

For presently-achievable laser intensities, period-four transition metals such as Cu are optimally situated for the efficient acceleration mechanism described in this work, in which ionization of the L-shell and acceleration occur simultaneously. The ionization energies of copper, shown in Fig. 1, illustrate the feature we aim to exploit in this work: there is a substantial gap in ionization potential that exists between different shells. This inherent property of elements can be used to filter a particular group of high-Z ions from a specific atomic shell and accelerate them selectively. For example, the ionization potential for Cu^+20^ (the potential to be overcome to remove the 20^th^ electron, the one that fills the L-shell), is almost three times the ionization potential for Cu^+19^ (to remove the sole electron left in the M-shell). In the case of ionization caused by the electric fields that accelerate the ions, this dramatic difference directly translates into a similarly dramatic difference in the field strength required to generate Cu^+19^ and Cu^+20^. We can thus expect the L-shell ions to appear only in the regions where the field is sufficiently strong such as in the laser focal spot. On the other hand, Cu K-shell ions have ionization potential too large for field ionization with existing lasers. Thus, in the focal spot one may only expect ions with valence L-shell or a full K-shell (20 ≤ Z ≤ 27). Thus, ions are selected both spatially and with a relatively narrow spread in Z. As a result, high-Z ions should experience a significantly stronger acceleration than the majority of their low-Z counterparts because of their location, which should lead to generation of energetic, low-emittance, high-Z ion beams. Moreover, the narrow range of Z can potentially lead to monoenergetic beams.

Because of this feature, spatially-resolved detection of high-Z ions in experiments with laser-irradiated targets can be used to gain valuable and otherwise inaccessible information about the accelerating field. By combining these measurements with kinetic simulations, information about the strength and structure of accelerating fields can potentially be extracted. Therefore, the discussed ionization effect is much more than a “control knob” for altering the ion spectrum, as it can also be used as a diagnostic tool for characterizing accelerating fields.

In what follows, we examine acceleration and simultaneous field ionization of copper ions in ultra-thin targets (20–150 nm thick) irradiated by a laser pulse with intensity of 1 × 10^21^ W/cm^2^. Our analysis is performed using two-dimension particle-in-cell (PIC) simulations. In line with our expectations, strong accelerating electric fields that are driven by the laser pulse of this intensity at the rear side of the target generate localized bunches of energetic ions with 20 ≤ Z ≤ 27. We also find that the thinnest target (20 nm) experiences a push by the laser consistent with the RPA mechanism that can further increase the ion energy gain, as the accelerating fields follow the accelerated ions. The effective ionization injection of high-Z ions into a strong accelerating field offers a promising mechanism for the production of quasi-monoenergetic, few-charge state, high-Z ion bunches rather than wide energy and charge state distributions.

In this paper, the study of the laser-target interaction is carried out in the regime most closely related to the TNSA. In the conventional TNSA regime, a laser pulse illuminates a target, transferring its energy to the target electrons. These heated electrons pass through the target and generate a strong charge-separation electric field at the target rear, which subsequently accelerates ions off the surface of the target. This highly idealized picture is often challenged in experiments and PIC simulations, which see a significant influence of the target thickness on the acceleration process. One of the main reasons for the thickness dependence in the case of ultra-thin targets is that the laser-driven ion acceleration is no longer exclusively in the TNSA regime, as it also starts to exhibit features of RPA or Coulomb Explosion^24^. For example, previous experiments and simulations have shown that the energy of accelerated gold ions can be boosted with targets thinner than 50 nm by inducing a Coulomb explosion^38^.

## Results

### Modeling approach

**Figure 2:**
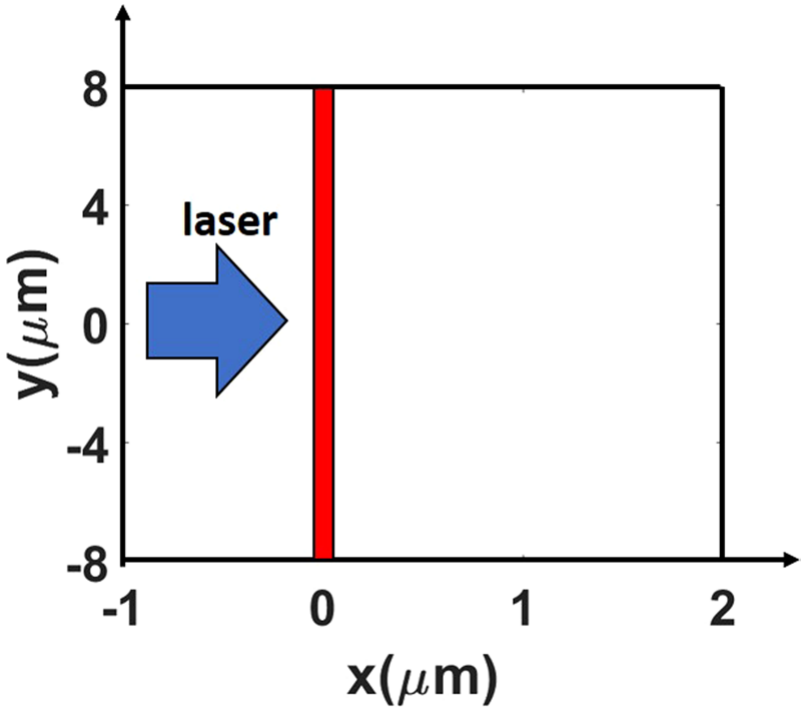
Schematic setup used in 2D PIC simulations of laser-irradiated ultra-thin targets. The box represents the full simulation domain. The target placement is shown in red, whereas the arrow indicates the direction of the incoming laser pulse. Note that the figure is not drawn to scale.

We use a fully-relativistic PIC code EPOCH^39^ to model the interaction of a high intensity (10^21^ W/cm^2^) ultra-short (35 fs) laser pulse with thin copper targets (20–150 nm) in order to further investigate key features of the ion acceleration regime where field ionization plays a major role. Our two-dimensional setup is represented in Fig. 2. The targets are irradiated by a linearly polarized high-intensity laser pulse (with wavelength of 800 nm) that horizontally enters the computational domain from the left side and focuses at x = 0 µm with a spot size of 4 µm. To provide a convenient point of reference, we define the time t = 0 fs as the moment when the laser intensity would reach its peak value in the focal plane in the absence of a target.

### Field ionization of Cu ions

Our primary interest is the interplay between ion acceleration and concurrent ionization by electric fields induced in the vicinity of the target surfaces. To appropriately simulate this, we use a Monte Carlo ionization algorithm that has been implemented in the EPOCH code^39^. The algorithm uses probabilities for field ionization that account for barrier suppression and tunneling ionization, using a generalized tunneling formula due to Ammosov, Delone, and Krainov (ADK)^40^ based on earlier work by Perelomov, Popov, and Terentev^41–43^. Multi-photon ionization is included for completeness, but its impact is negligible in the regime of interest. The energy loss caused by field ionizations is taken into account by a current density correction^39,44,45^, as described in the *Methods* section. Other atomic processes that occur between electrons, ions and photons are not considered in our simulations, and their effects are discussed later in the section *Other atomic processes*.

In our simulations, Cu ions are primarily ionized due to the tunneling and barrier suppression at the considered laser intensities and sheath field strengths. The ionization is modelled as a probabilistic process rather than a deterministic one. This means that ionization of an ion exposed to a strong electric field directly depends on the duration of the exposure. This is particularly true for the transitions within each shell, because the corresponding values of the ionization potentials are very close to one another (see Fig. 1).

In order to provide some context for the discussion that will follow, we have carried out several simulations where a low density (8.4 × 10^8^ atoms/m^3^) Cu foil was placed in a uniform, steady applied electric field. The goal here is to illustrate how ionization fraction evolves over time, depending on the amplitude of the applied electric field. The range of field amplitudes most relevant to the simulations presented later is between 1 × 10^13^ and 1 × 10^14^ V/m. The upper limit is set by the laser electric field. The laser field amplitude peaks at 8.6 × 10^13^ V/m (which corresponds to the peak intensity of 9.8 × 10^20^ W/cm^2^) if no target is present. The combination of the incoming and reflected fields increases the amplitude of the transverse electric field at the front of the target to about 1.65 × 10^14^ V/m. The sheath electric field generated by electrons at the rear side of the target peaks at about 4.5 × 10^13^ V/m, while exceeding 1 × 10^13^ V/m over a significant portion of the rear side. The structure of the sheath field will be discussed in detail in the following section, so here we only focus on the impact that fields with these amplitudes have on the ionization state of Cu ions.

**Figure 3:**
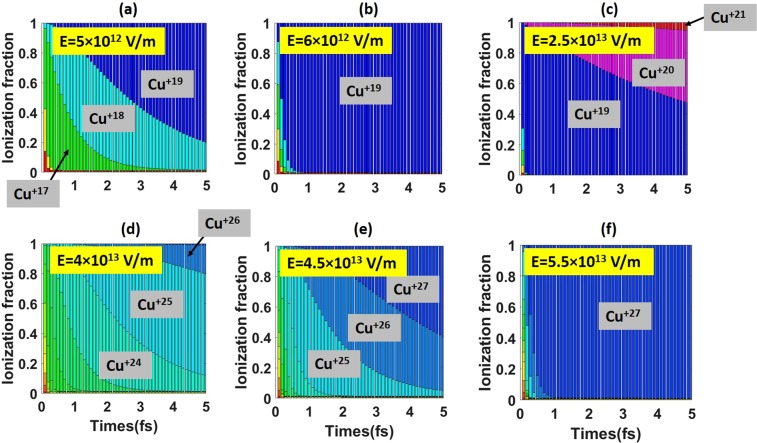
Calculated ionization fraction as a function of time for a low-density Cu foil in a constant applied electric field. The applied field strength ranges from 5 × 10^12^ to 5.5 × 10^13^ V/m.

As illustrated in Fig. 1, there is a significant gap between ionization potentials for Cu^+19^ and Cu^+20^, which represents a transition between the M-shell and the L-shell. This implies that there is wide range of electric field strengths, which would quickly ionize copper to Cu^+19^ while producing almost no Cu^+20^. It can be seen in Fig. 3 that this range roughly corresponds to field strengths in the range 5 × 10^12^ V/m < E < 2.5 × 10^13^ V/m. For example, all Cu ions are turned into Cu^+19^ in less than 1 fs in an applied field of 6 × 10^12^ V/m (see Fig. 3b). The implication for our simulations is that we should expect Z ≥ 19 throughout the Cu targets that we simulate.

Similar logic applies when considering the gap between the L-shell and the K-shell that corresponds to a transition from Cu^+27^ to Cu^+28^. For a field with an amplitude above 5.5 × 10^13^ V/m, the Cu ionization state quickly saturates at Z = 27, as seen in Fig. 3f. A much stronger field is required to achieve Z = 28. The field amplitude in our simulations remains below 1.65 × 10^14^ V/m, so we do not expect the laser-target interaction to generate any Cu^+28^ ions through field ionization.

As evident in Fig. 3, the time evolution of copper ionization states is qualitatively different if the field strength is between 2.5 × 10^13^ and 4.5 × 10^13^ V/m. By changing the field by less than a factor of two, ionization can reach the next atomic shell and the maximum Z can be changed from 20 to 27. This is due to the fact that the ionization potentials are very close to each other within the L-shell (20 ≤ Z ≤ 27). The proximity of the ionization levels means that the corresponding probabilities for a given electric field are also very similar. That is why the number of Cu^+25^ ions in Fig. 3d is comparable to the numbers for Cu^+24^ and Cu^+26^. This is also the reason why the ionization fraction continues to evolve without visible saturation on a time scale of multiple femtoseconds (see Fig. 3d,e).

At the same time, the proximity of the ionization levels in the L-shell can still be roughly interpreted as having high sensitivity to the electric field if the electric field is in the range between 2.5 × 10^13^ and 4.5 × 10^13^ V/m. For example, by slightly increasing the field from 4 × 10^13^ V/m to 4.5 × 10^13^ V/m we can completely eliminate Cu^+24^, provided that the ions are exposed to the applied field for several femtoseconds. Based on these estimates, the window of the electric field strength is very narrow, and it may seem impractical to achieve such control of the ionization states. However, the dependence on the laser intensity is not as sensitive due to its weak relation with the sheath electric field strength. According to Mora’s plasma expansion theory^46^ and the ponderomotive scaling of hot electron temperature to the laser intensity^47^, the TNSA sheath field strength *E*_*x*_ is approximately proportional to the square root of the hot electron temperature *T*_*e*_, which scales as the square root of the laser intensity *I*. Therefore, *E*_*x*_ scales as *I*^*1/4*^, meaning that a rather large window of laser intensity corresponds to a narrow window of *E*_*x*_. The laser intensity can be conveniently controlled in the experiments by changing the laser pulse energy, duration, or spot size. This offers an attractive mechanism to selectively generate very localized distributions of high-Z copper ions. How this can be achieved in a self-consistent setup is explored in the following section.

### “Ionization injection” - concurrent ionization and acceleration of high charge state ions

**Figure 4:**
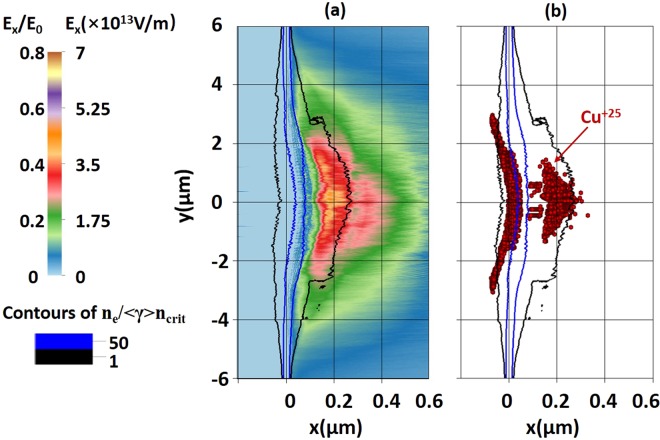
Snapshots of the longitudinal electric field (E_x_ > 0 only) and Cu^+25^ ions taken at t = 0.5 fs for a target whose initial thickness was 20 nm. Here t = 0 fs corresponds to the time when the pulse peak reaches the target front surface. The black and blue curves are the contours of the electron density averaged over one laser period. The field is normalized to E_0_ = 8.6 × 10^13^ V/m, which is the peak laser electric field at the focal spot when the target is not present.

Now we present the simulation results of solid-density Cu foils of several thicknesses irradiated by a laser. The details are given in the Methods section. The response of the thinnest considered Cu target (20 nm) to the intense laser pulse with parameters listed in the Section 1 is shown in Fig. 4. The leading edge of the laser pulse quickly ionizes the copper atoms, turning the target into an opaque plasma. Here the opaque condition can be estimated with Vshivkov’s formula^48^:

$${n}_{e}/({a}_{0}{n}_{crit})=\lambda /(l\pi ),$$

where n_e_ is the electron density, *λ* = 800 nm is the laser wavelength, *l* = 20 nm is the target thickness and ${a}_{0}=0.85{(\frac{I{\lambda }^{2}}{{10}^{18}W/c{m}^{2}\mu {m}^{2}})}^{1/2}=21$ is the dimensionless amplitude of the laser with intensity *I* = 9.8 × 10^20^ W/cm^2^. It gives ${n}_{e}=4.8\times {10}^{29}/{m}^{3}$, showing that the foil becomes opaque to the laser when the electron density *n*_*e*_ exceeds this value. Considering the copper atom density n_a_ = 8.4 × 10^28^/m^3^, the opaque condition only requires a charge state Z ~ 6, which can be reached due to field ionization in the laser field, according to our discussion in the previous section. Indeed, our simulation results indicate that only a very small portion of the laser energy is transmitted through the target. The rest of the pulse is in part reflected and in part absorbed at the laser-irradiated side of the target. The black and blue curves in Fig. 4a,b mark the contours of the relativistically adjusted critical density n_*_ and 50n_*_. We define n_*_ as the classical critical density n_crit_ multiplied by a local relativistic gamma-factor, <γ>, that has been averaged over the laser period and over the electron population in each cell. The contour of n_*_ describes the density beyond which the laser intensity begins to decay in space, and the contour of 50n_*_ approximately represents the shape of the target foil, which has been pushed and bent during the interaction with the laser pulse.

The absorbed laser energy is converted into kinetic energy of the target electrons. The movement of the laser-heated electrons induces charge-separation and, as a result, generates a sustained electric field normal to the target surfaces. Figure 4a shows a snapshot of the positive longitudinal electric field at t = 0.5 fs. The field has the highest amplitude close to the axis of the laser pulse. The field amplitude falls off in the transverse direction on a spatial scale roughly determined by the size of the laser focal spot. This longitudinal field gradually accelerates the ions off the rear side of the target.

Simultaneously with the acceleration, the electric field is also able to ionize the copper ions. The longitudinal electric field at the rear side is much weaker than the transverse laser electric field at the laser-irradiated side, which creates a very different environment for the field ionization. At the front, the total electric field is so strong that it quickly ionizes copper ions to Z = 27. The field strength is however not sufficient to overcome the jump in the ionization potential between the L and K shells to produce Cu^+28^. As a result, the front side is primarily populated by Cu^+27^ ions that are broadly distributed without much spatial localization.

At the rear side, the electric field peaks at about 4.5 × 10^13^ V/m, which is sufficient to quickly generate ions with Z = 20, but the ionization to significantly higher Z states in the L-shell is a gradual process that occurs on a time scale comparable to the ion acceleration time. This process is illustrated in Fig. 3d,e. As a result, the transverse and longitudinal localization of the electric field shown in Fig. 4a directly translates into producing very localized populations of Cu^+24^ and higher Z states. Figure 4b shows a scatter plot of Cu^+25^ which directly illustrates this point. A wide lateral band at the laser-irradiated side is generated by the field of the laser, whereas a more localized population at the rear side is generated by the sheath electric field. There is a clear correlation between the size of the region with the strong sheath field and the area occupied by the Cu^+25^ ions.

One can draw an important conclusion from the simulation presented in Fig. 4. A sheath electric field can create spatially localized populations of high-Z ions if its strength is close to the strength required for barrier suppression to start ionizing the first level beyond a filled shell. In the case of Cu ions, the desired field range to produce localized ions in the L-shell is from 2.5 × 10^13^ to 4.5 × 10^13^ V/m.

**Figure 5:**
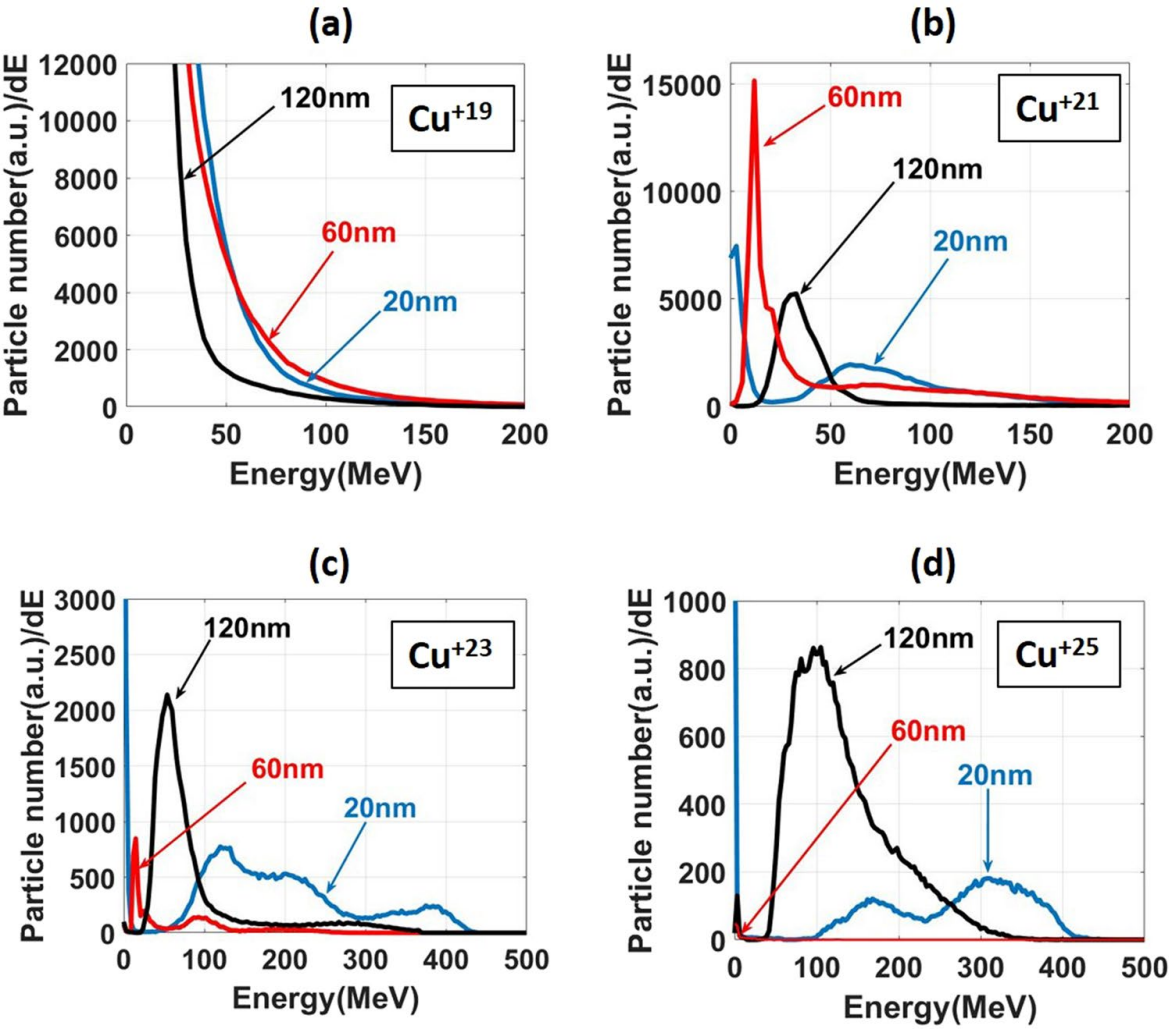
Energy spectra of Cu^+19^, Cu^+21^, Cu^+23^ and Cu^+25^ ions for target thicknesses of 20, 60 and 120 nm at the end of the simulation (t = 17 fs).

The localization of the high-Z ions in a region with a strong longitudinal electric field means that these ions will experience a much stronger acceleration than ions on the periphery, as the target expands. Figure 5 presents ion spectra at the end of the simulation when the ionization process is effectively over and the localization of L-shell ions is already locked in. Figure 5a shows the energy distribution of Cu^+19^ for three different target thickness: 20, 60, and 120 nm. All three curves are monotonically decreasing, which is the dependence typically expected from TNSA. The underlying reason is a broad spatial distribution of Cu^+19^ ions over a region with a wide range of electric field values. As discussed in the field ionization study, Cu^+19^ is reached by removing the last M-shell electron at just 6 × 10^12^ V/m in less than one laser period (see Fig. 3b). That is why Cu^+19^ ions are plentiful throughout the target, including the rear surface where the acceleration takes place.

Figure 5 also shows the energy spectra for Cu^+21^, Cu^+23^, and Cu^+25^, and these are qualitatively different from the spectrum of Cu^+19^. These spectra have very pronounced peaks at high energies. This is a direct consequence of the already discussed localization of these ions in defined regions corresponding to strong accelerating fields. In other words, the high-Z ions undergo an “ionization injection”. The mechanism is quite different from that of electron ionization injection in laser wakefield acceleration^49–52^ but the two have something in common: ionization at both the right time and location which can create the right conditions for generating a co-moving bunch of particles. Since the high-Z ions on the rear are injected into a strong electric field that imparts to them a non-negligible energy gain, it is not surprising that many of the high-Z ions’ spectra are missing the usual low energy ions. This effect gives a quasi-monoenergetic characteristic to ion bunches for Cu^+21^, Cu^+23^, and Cu^+25^, as opposed to the broad exponentially decaying spectrum for Cu^+19^.

### Target thickness effect

In the previous section, we demonstrated that a sheath electric field in a certain range of amplitudes can induce what can be viewed as an ionization injection of high-Z ions into a strong accelerating field. This is the trend that all of the considered target thicknesses have in common. However, a closer inspection of the peaked ion spectra in Fig. 5b,c,d reveals that the 20 nm target performs consistently better than the rest of the simulated targets, generating higher energy ion bunches. The energies corresponding to the peaks of the 20 nm target are at least a factor of two higher than the energies from the 60 and 120 nm targets. In what follows, we examine the cause for this enhanced performance.

**Figure 6:**
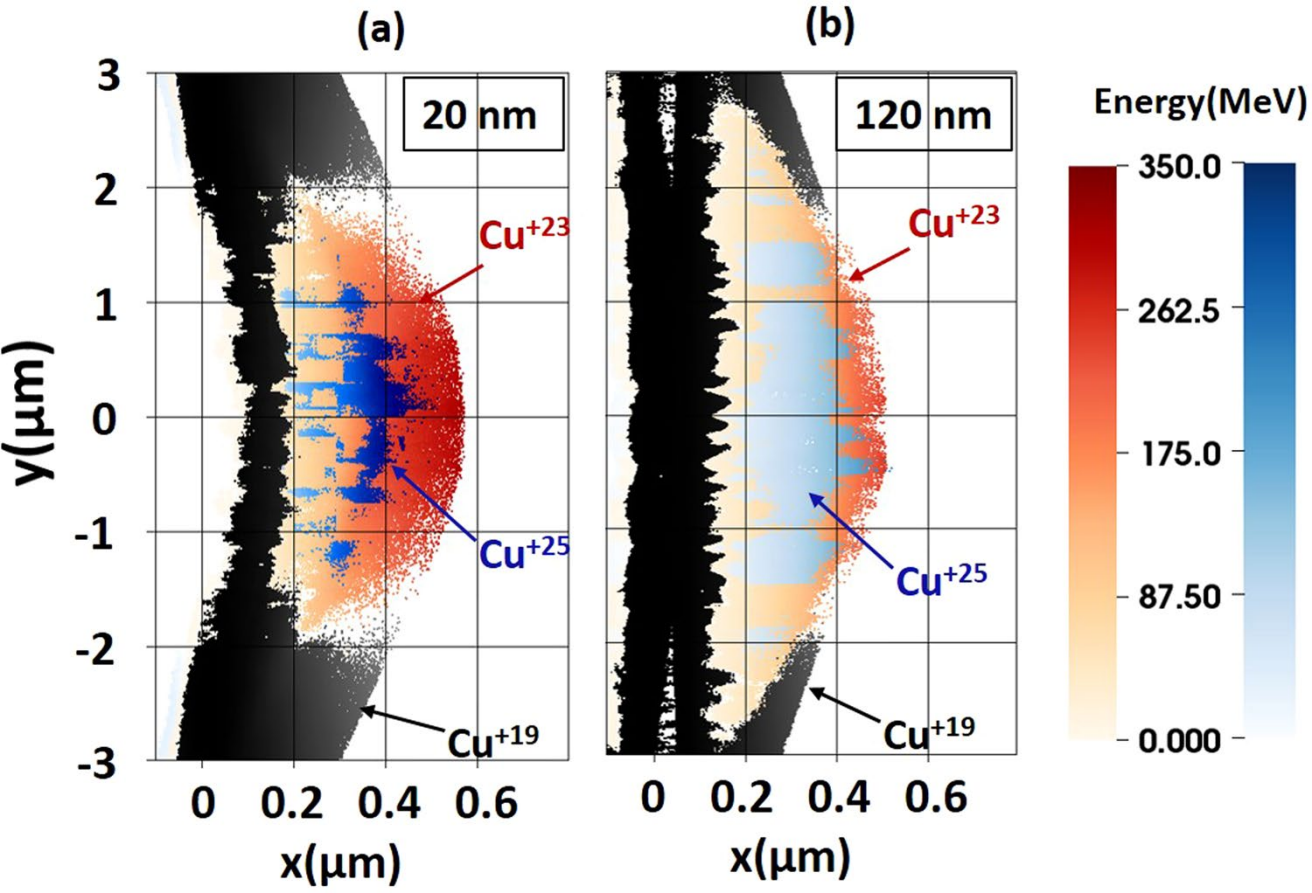
Scatter plots of Cu^+19^, Cu^+23^, and Cu^+25^ ions at t = 10 fs for targets whose initial thickness was (**a**) 20 and (**b**) 120 nm. Different colors show the three states, and the hue for Cu^+23^ and Cu^+25^ ions represents their kinetic energies.

Figure 6 shows scatter plots of Cu^+19^, Cu^+23^, and Cu^+25^ ions for two different target initial thicknesses of 20 and 120 nm. As already pointed out, the thinner 20 nm target produces higher energy ions. This is clearly visible comparing the hue of Cu^+23^ and Cu^+25^ ions in the two panels. It is also evident from looking at Cu^+19^ ions that the thinner target has experienced a significant push by the laser in the forward direction. As compared to the Cu^+19^ ions of the 120 nm target, the Cu^+19^ ions on the laser-irradiated side of the 20 nm target appear to have traveled more than 100 nm in the forward direction.

In order to confirm that the laser pulse can indeed provide a significant push to the 20 nm target, we assume that the underlying mechanism is the radiation pressure acceleration. The optimal target thickness for RPA can be calculated with the following formula^48^

$${l}_{opt}={a}_{0}\frac{\lambda }{\pi }\frac{{n}_{crit}}{{n}_{e}}$$

where *l*_*opt*_ is the optimal target thickness. In our simulation, *n*_*c*_ = 1.75 × 10^27^/m^3^, n_e_ = 1.6 × 10^30^/m^3^ (calculated by the initial copper atom density and the dominant charge state of 19) and *λ* = 800 nm, *I* = 9.8 × 10^20^ W/cm^2^ and a_0_ = 21.3. From these parameters, the optimal target thickness is *l*_*opt*_ = 6 nm. By comparison, the target thickness of 20 nm is three times *l*_*opt*_, showing that the 20 nm thick target may partially fall on the RPA domain while the 60 and 120 nm targets are 10x and 20x too dense and most likely in the TNSA regime.

We can then estimate the target displacement by employing the results of refs^53,54^. In the non-relativistic regime, the maximum ion energy is given by the following formula:

$${E}_{Cu}=8\times {({10}^{11}/{N}_{tot})}^{2}({m}_{p}/{m}_{Cu}){({E}_{las}/1J)}^{2}\,MeV$$

where *N*_*tot*_ is the total number of ions being accelerated, *m*_*p*_ is the proton mass, *m*_*Cu*_ are the mass of a copper ion, and *E*_*las*_ is the total energy in the laser pulse. By *t* = 0, the ions should already acquire energy of 13 MeV, which corresponds to a velocity of 0.02*c*, where *c* is the light speed. Taking the travel time to be half of the laser pulse duration (~17 fs), we find that the resulting target displacement should be about 100 nm. This is in good agreement with our simulation results shown in Fig. 6a, where the target is visibly bent and the Cu^+19^ ions located at y = 0 µm have been displaced in the forward direction (to the right) by roughly 100 nm. For the target with an initial thickness of 120 nm, the number of ions that the laser pulse has to push is six times higher, so the expected displacement is only 17 nm. Such a small displacement is difficult to identify in the fully self-consistent PIC simulation whose results are shown in Fig. 6b. The difficulty is caused by the target expansion that happens at the same time and, in the case of a thicker target, it is the dominant effect at the laser-irradiated surface.

We can now conclude that the displacement at the front side of the target with an initial thickness of 20 nm is not negligible compared to the thickness of the target following its expansion. This clearly demonstrates that the expansion caused by the sheath field and the forward acceleration caused by the laser pulse are comparable effects observable only for the thinnest targets considered here.

### Tracking the field dynamics and structure

**Figure 7:**
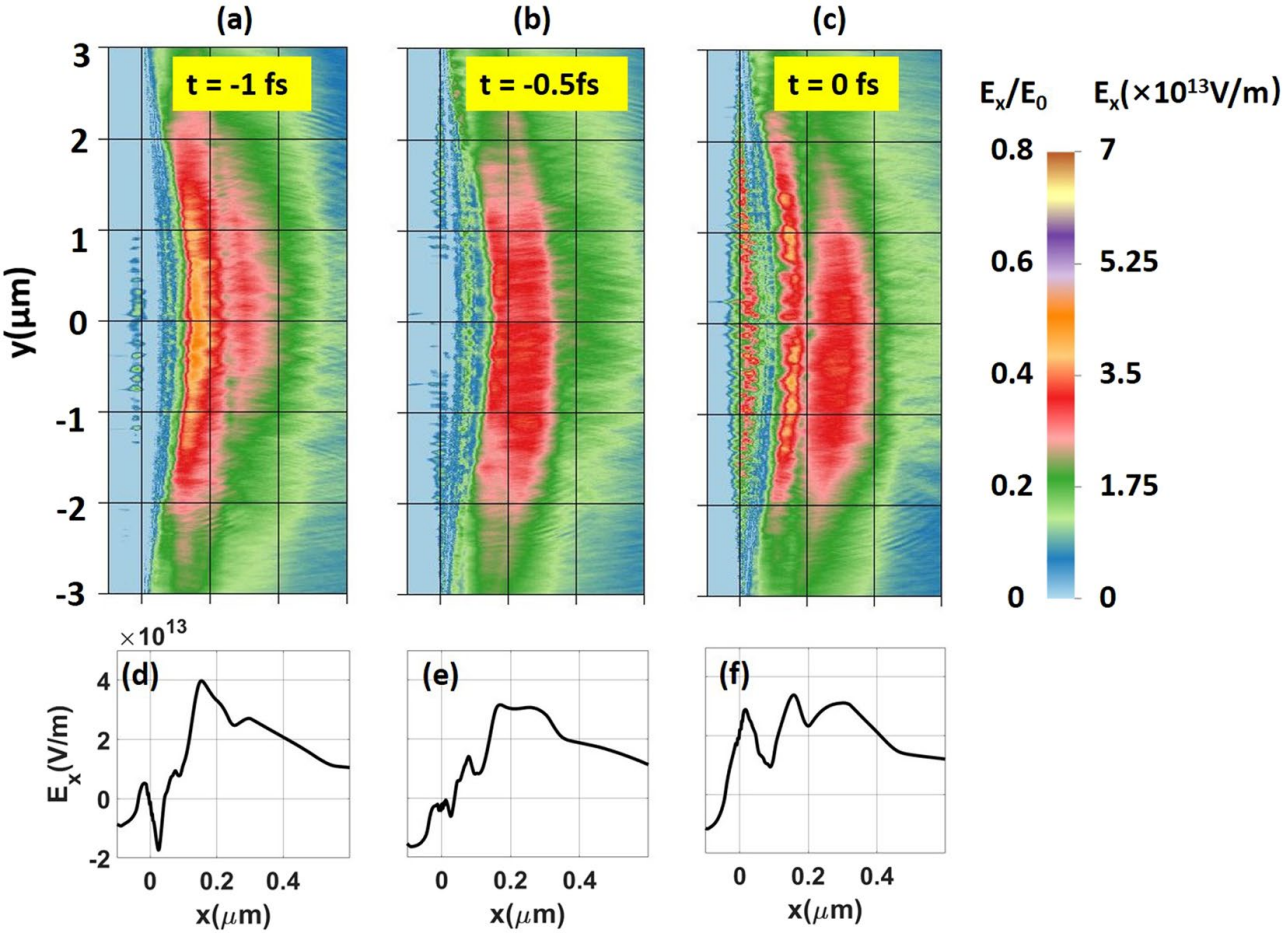
Snapshots of the longitudinal electric field at the rear side of the 20 nm target at t = −1 fs,−0.5 fs, and 0 fs. The lineouts are transversely averaged over the interval −1 µm < y < 1 µm.

In order to relate the differences in spectrum with acceleration mechanisms, we examine the time evolution and structure of the accelerating sheath field at the rear side of the irradiated targets. Figure 7 shows the snapshots of the longitudinal electric field taken just 0.5 fs apart (t = −1, −0.5 and 0 fs). There are two striking features that distinguish this field from the conventional TNSA field generated at the surface of a thick target. In our case, the field has a two-band structure and this structure, including the location sheath field maximum, oscillates at roughly twice the laser field frequency. Plots of electron density (not shown here) reveal that in addition to the heating of target electrons, the laser pulse also accelerates a part of the electron population in the form of thin sheets, similar to what is observed in the experiments of high-intensity laser-solid target interaction^55^. As the electron sheets move through the expanding plasma at the back of the target, they locally enhance the charge-separation field and produce the structures observed in Fig. 7. It is worth noting that an oscillating longitudinal field (~10^13^ V/m) is also generated on the target front surface [near x = 0 in Fig. 7]. This field stays in the thin skin layer to balance the ponderomotive force of the laser^56^. When RPA is dominant, this field can be extended over most of a thin target. However, it is not the case in our simulation since the target thickness is three times the optimal thickness for RPA. Therefore, this field only stays in the thin skin layer, and does not contribute much to the ionization process.

**Figure 8:**
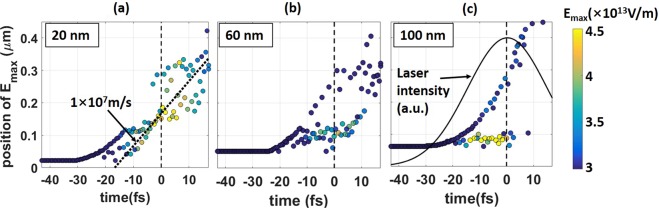
Location of the strongest electric field as a function of time for three different target thicknesses. The amplitude of the field is color-coded. The dashed lines mark the time when the laser peak intensity reaches the target. The dotted line in the left panel represents the slope corresponding to 1 × 10^7^ m/s.

In order to determine the long-term trend in the evolution of the sheath field structure, we have tracked the location of the maximum longitudinal electric field, E_max_, from t = ~40 fs to t = 17 fs. Figure 8 shows the results of the tracking for three target thicknesses. The color here indicates the amplitude of E_max_. Early in the laser pulse (around t ~ −15 fs), the field structure appears to be similar for all three targets. However, its subsequent evolution strongly depends on the target thickness. In the case of the target with an initial thickness of 120 nm, two separate branches develop in Fig. 8c at t > −13 fs. The lower branch has a much stronger peak field and it represents a region with a sharp density gradient. The strong field remains confined to the target rear-side. The upper branch corresponds to the relatively weak electric field at the very front of the expanding ion population. In contrast to that, the 20 nm target has just a single branch in Fig. 8a, where the jitter reflects the time-varying structure that we have discussed above and shown in Fig. 7. In this case, the strong field moves forward instead of being fixed. We will now show that this electric field is co-moving with the target pushed forward and bulk ions at the previously estimated velocity of ~0.02c.

Indeed, the strong field generated by the 20 nm target moves in the direction of the longitudinal ion acceleration at a velocity of approximately 10^7^ m/s, as indicated by the dotted line in Fig. 8a. The energetic bunches that the 20 nm target generates have comparable longitudinal velocities. For example, the Cu^+21^ spectrum in Fig. 5b peaks at 60 MeV. The corresponding velocity of a copper ion is 1.3 × 10^7^ m/s. This means that the strong sheath field is able to accelerate high-Z ions for much longer than in the case of thicker targets where the strong field is not moving in the longitudinal direction (see Fig. 8b,c). We then conclude that the forward directed movement of the thin 20 nm target is the primary reason for the enhancement of the high-Z ion spectra shown in Fig. 5. It is important to note that the RPA does not contribute to the quasi-monoenergetic spectra from 50 to 400 MeV shown in Fig. 5 because the 20 nm target velocity caused by the radiation pressure corresponds to an energy below 20 MeV. It can be seen in the Cu^+21^ spectrum in Fig. 5b.

### Other atomic processes

The focus of our work is on the field ionization process and its critical role in generating localized bunches of high-charge ions. However, there are other ionization processes that can potentially disrupt the described localization. In the Supplementary document, we assess the impact of two such processes, collisional ionization and photoionization, to formulate the applicability of our analysis that assumes that the field ionization is the dominant process. The Supplementary document also examines the impact of pre-pulses that are inherent to short pulse lasers in experiments. The conditions that allow one to neglect the target expansion is provided.

Ion-ion charge exchange may take place during the propagation of the accelerated ions. However, it should not affect our conclusions because most products of the ion-ion charge exchange of L-shell ions are still L-shell ions. In our simulations, all the L-shell ions are localized near y = 0 ± 2 µm and mixed together without any lower charge state (Cu^+19^) nor higher charge state (Cu^+28^) [Fig. 6]. Lower charge ions (Cu^+19^) are mainly at greater radii and have lower energy than L-Shell ions. Hence, even if the ion charge exchange occurs in the ion bunch, it mostly occurs among L-shell ions and redistributes their numbers. For those ion exchanges that may result in Cu^+19^, the ion Cu^+20^ must be involved. Since Cu^+20^ ions are located on the periphery of the generated L-shell ion bunch, these ion exchanges should only cause a little mix of Cu^+19^ on the periphery of the bunch. Therefore, we think the effects of ion charge exchange do not modify our key conclusion relying on the spatial localization of the L-shell ions.

### Summary

We have examined the process of ion acceleration in the interaction of an ultra-short (35 fs) intense laser (10^21^ W/cm^2^) and ultra-thin (20‒150 nm) copper targets using kinetic particle-in-cell simulations. We find that high-Z Cu^+20‒27^ ions are ionized and accelerated mainly by the sheath electric field through the target normal sheath acceleration (TNSA) mechanism on the target rear surface. The high-Z ions are produced only in a very localized region due to a significant gap between the M- and L-shells’ ionization potentials and can be accelerated by strong, forward-directed sections of the field. Such an “ionization injection” leads to well-pronounced bunches of energetic high-Z ions. We also find that the TNSA acceleration can be further enhanced in the case of the 20 nm target due to the target movement caused by the radiation pressure acceleration.

We focus our study exclusively on copper in this paper, but our conclusions are easy to generalize to targets of different composition and may be applied to a number of other cases. By choosing a lower atomic number target material with K-shell ionization potential suitably matching the laser intensity, beams of predominately one or two charge states with spectral peaks may be possible. It is also conceivable that the “s” subshell of higher atomic number materials (e.g. the first subshell of the Ag M-shell) could be targeted.

## Methods

### Simulation setup

The simulation domain is 3 µm long and 16 µm wide, with an initially neutral solid-density copper target centered at x = 0 μm. Simulations are performed for targets that are 20, 40, 60, 80, 100, 120, and 150 nm thick. We use 500 cells per µm along the x-axis and 250 cells per µm along the y-axis. The time step in the simulations is 5.7 × 10^−3^ fs. These parameters are chosen based on a convergence benchmark made to ensure that the results presented in the manuscript are not sensitive to the cell size. Uniform ultra-thin targets with an atom density n_a_ = 8.4 × 10^28^/m^3^ are initiated using 200 macro-particles per cell. The focal plane of the pulse is located at x = 0 µm, with the x-axis also being an axis of symmetry for the laser pulse. The laser wavelength is 800 nm and the peak intensity in the focal plane (when the target is not present) is 9.8 × 10^20^ W/cm^2^. The transverse intensity profile is Gaussian with a 4 µm focal spot size, which is defined as the full width at half maximum of the intensity (FWHM). The temporal profile of the pulse intensity is also Gaussian, with a pulse duration of 35 fs at FWHM. We use open boundary conditions for fields and particles to eliminate reflections.

### Field ionization

The field ionization packages in EPOCH, including barrier suppression ionization, tunneling ionization and multiphoton ionization, can be described as follows. In every time step, the electric field strength on each particle is calculated. Depending on the field strength, the ionization model is determined, and the ionization rate is attained by a probabilistic calculation based on the multi-photon or tunneling ionization or barrier suppression ionization theory.

Multiple ionizations during one time step are accounted for by sampling the time of the ionization t_ionize_ based on the ionization rate. If t_ionize_ is smaller than the time step, this particle goes back to the ionization loop, and it may be further ionized. The ionization time is accumulated to t_ionize_. This process is repeated until t_ionize_ exceeds the time step.

To account for energy loss caused by field ionization and conserve energy in the code, a correction is made to current density based on Poynting’s theorem. For tunneling and barrier suppression ionization, the energy loss from the field corresponds to the sum of ionization energy of all the ionization steps that occur in each time step. For multi-photon ionization, the energy loss is the total energy of absorbed photons. In our simulations, the energy loss by the ionization is ~10^−6^ of the input laser energy. The details of the field ionization in EPOCH can be found in Section 3.2 of ref.^39^.

## Supplementary information

Supplementary Information
