# Supplementary material for: Ionization injection of highly-charged copper ions for laser driven acceleration from ultra-thin foils
Source: Sci Rep. 2019 Jan 24;9:666. doi: 10.1038/s41598-018-37085-6 (PMC6345865; doi:10.1038/s41598-018-37085-6)
Supplement: Supplementary file 1 — Supplementary Information [file 41598_2018_37085_MOESM1_ESM.docx]

**Supplementary Material**

**Ionization injection of highly-charged copper ions for laser driven acceleration from ultra-thin foils**

**Jun Li**^1^**, Alexey V. Arefiev**^1,2^**, Stepan S. Bulanov**^3^**, D. Kawahito^1^, Mathieu Bailly-Grandvaux**^1^**, George M. Petrov**^4^**, Christopher McGuffey**^1^**, and Farhat N. Beg^*^**^1,2^

^1^Center for Energy Research, University of California San Diego, La Jolla, CA, 92093, USA

^2^Department of Mechanical and Aerospace Engineering, University of California San Diego, La Jolla, CA 92093, USA

^3^Lawrence Berkeley National Laboratory, Berkeley, CA, 94720, USA

^4^Naval Research Laboratory, Plasma Physics Division, Washington, DC 20375, USA

*fbeg@ucsd.edu

The focus of our work is on the field ionization process and its critical role in generating localized bunches of high-charge ions. However, there are other ionization processes that can potentially disrupt the described localization. In what follows, we assess the impact of two such processes, collisional ionization and photoionization, to formulate the applicability of our analysis that assumes that the field ionization is the dominant process.

Collisional ionization

Collisional ionization by electrons takes place not only during the main pulse, but also during its pre-pulse that can be subdivided into two distinct stages. The first stage originates from an amplified spontaneous emission (ASE) background and typically has a ns time scale. The intensity of the ASE pre-pulse can be reduced to 10^-11^ of the main pulse intensity by utilizing an optical-parametric CPA (OPCPA) technique [1]. ASE cleaning can also utilize other techniques, such as cross-polarized wave (XPW) generation [2], double CPA [3], frequency-doubling post compression [4], and absorbers/plasma mirrors [5,6]. In the second stage, the laser intensity rises from the ASE background to the foot of the main pulse on a ps time scale. The characteristic intensity is typically 10^-5~6^ of the peak intensity of the main pulse. This ratio can be reduced to 10^-7~8^ with the above mentioned techniques. The ionization processes during these two stages are different and need to be discussed separately.

Collisional ionization during the pre-pulse

In order to evaluate the ionization during the ns-long pre-pulse, we use the 1D radiation-hydrodynamics code HELIOS [7] that includes collisional ionization. We consider a 100 nm thick Cu foil irradiated by a laser pulse with an intensity of 10^10^ W/cm^2^. The target undergoes very low ionization, with the mean charge not exceeding Z = 4 [Fig.1 (b)]. The target surface expands because of the laser heating, but the peak density remains close to 80% of the initial target density [Fig.1 (a)], indicating that the target remains almost intact at the considered intensity. Two more simulations were performed with different intensities. At 10^9^ W/cm^2^ (contrast ratio <10^-12^), the ionization state stays low (Z = 4) at the end of a 1 ns long simulation. The peak density of the target remains unchanged and the expansion is negligible [Table I]. At 10^11^ W/cm^2^ (contrast ratio <10^-10^), the ionization state is similar to that in the other two simulations. However, the target expansion becomes significant and the peak density drops to 4% of the initial density [Table I]. This transition agrees well with a fluence threshold of 10 J/cm^2^ that has been confirmed in experiments with laser-irradiated Cu foils [8,9]. We conclude that that the ionization caused by the ASE pre-pulse is insignificant in the context of our study. However, the pre-pulse fluence must be below 10 J/cm^2^ in order for the target expansion to be negligible as well.


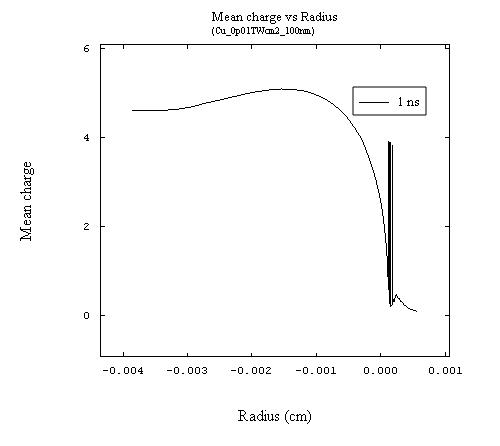

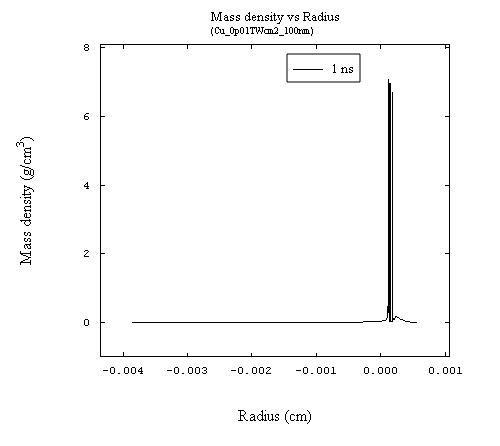


**-40**

**-20**

**0**

**x (µm)**

**Mean charge state Z**

**6**

**4**

**2**

**0**

**Mass density (g/cm^3^)**

**-40**

**-20**

**0**

**x (µm)**

**8**

**6**

**4**

**0**

**2**

**(b)**

**(a)**

***Figure 1****. (a) Mass density and (b) mean charge state Z after a 1 ns long interaction of a 10^10^ W/cm^2^ pulse with a 100 nm thick Cu foil. The laser-irradiated surface is initially located at x = 0. The laser pulse propagates in the positive direction along the x-axis.*

***Table I****. Effect of a ns-long pre-pulse on a 100 nm thick Cu target*

| Contrast | 10^-12^ (*I* = 10^9^ W/cm^2^) | 10^-11^ (*I* = 10^10^ W/cm^2^) | 10^-10^ (*I* = 10^11^ W/cm^2^) |
| --- | --- | --- | --- |
| Peak mass density ratio to solid copper density | 1 | 0.8 | 0.04 |
| Mean charge state Z | 4 | 4 | 5 |

The second stage of the pre-pulse corresponds to the rise in the laser intensity from the low intensity pre-pulse to the foot of the main pulse on a ps-time scale. For a contrast ratio of 10^-6^, the corresponding pre-pulse intensity for our parameters is *I* =10^15^ W/cm^2^. We again consider a 1D 100 nm thick Cu target. The laser-target interaction is modelled using a PIC code [10] that includes an electron impact ionization process described by the BEB model [11] in addition to the field ionization. After 1 ps, no ions with Z = 20 are produced by the pre-pulse [Fig.2 (a)] and the ionization is primarily caused by collisions [Fig.2 (b)].


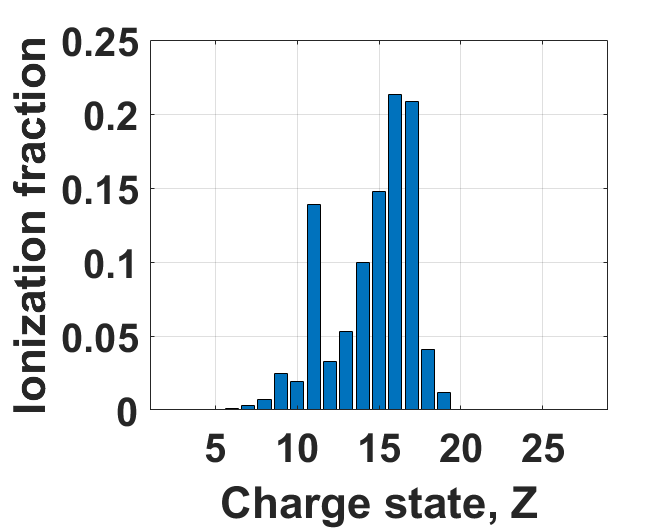

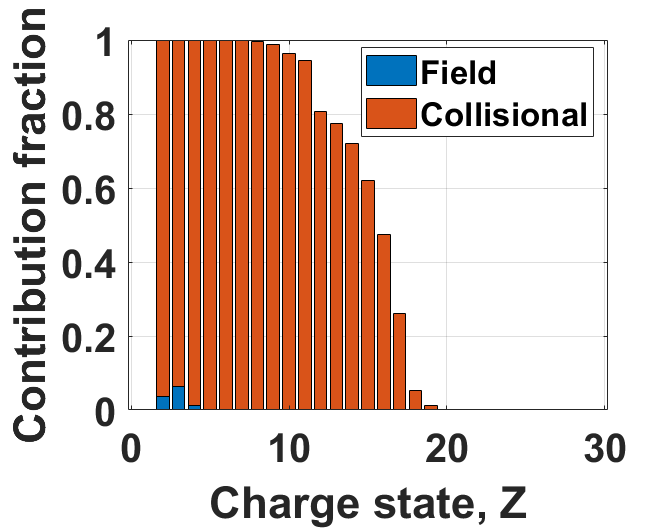

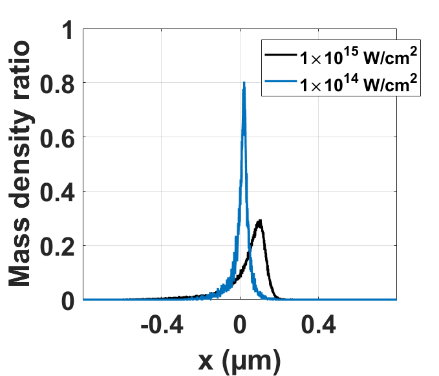


**(a)**

**(b)**

**(c)**

*Figure 2. (a) Ionization fraction for different charge states Z and (b) the corresponding relative contributions of field and collisional ionization processes. (c) Mass density profiles normalized by the solid Cu density after a ps-long laser-target interaction at two different pre-pulse intensities.*

Even though the L-shell electrons remain unaffected by the ps-long pre-pulse with *I* =10^15^ W/cm^2^, the energy delivered by the pre-pulse causes significant target expansion [see Fig.2(c)] compared to its initial thickness. The peak density drops to 30% of its original value during the expansion. Such an expansion is inconsistent with our assumption that the target remains intact prior to the arrival of the main laser pulse. Nevertheless, it is sufficient to reduce the pre-pulse intensity by a factor of ten, which would correspond to a contrast ratio of 10^-7^, in order for the expansion to become insignificant. This conclusion follows from an additional simulation with a peak intensity of *I* = 10^14^ W/cm^2^ [see Fig.2(c) and Table II]. We then conclude that our results are applicable for laser pulses with a contrast ratio of 10^-7^ for the ps-scale part of the pre-pulse. This contrast ratio is achievable with the help of the already existing techniques.

***Table II****. Effect of a ps-long pre-pulse on a 100 nm thick Cu target*

| Contrast | 10^-7^ (*I* = 10^14^ W/cm^2^) | 10^-6^ (*I* = 10^15^ W/cm^2^) | 10^-5^ (*I* = 10^16^ W/cm^2^) |
| --- | --- | --- | --- |
| Peak mass density ratio to solid copper density | 0.8 | 0.3 | 0.09 |
| Maximum charge state Z | 16 | 19 | 21 |
| Average charge state Z | 12.6 | 14.8 | 18.2 |

Collisional ionization during the main pulse and the subsequent ion acceleration process

In this section, we study the effects of the collisional ionization during the main pulse using a PIC code [10] that includes well-benchmarked collisional and field ionization modules. Two 1D simulations are performed: a simulation that accounts only for the field ionization process and a simulation that includes both field and collisional ionization processes. Their results are compared to determine the relative role of the collisional ionization.

The 1D simulations are initialized with similar physical and numerical parameters to the 2D simulations in the main text. The simulation domain is 3 µm long from x= -2 to 1 µm. The copper target with thickness of 20 nm is centered at x = 0 µm with neutral atoms and solid copper density. The laser is incident from x= -2 µm with wavelength of 800 nm and peak intensity of 9.8×10^20^ W/cm^2^. The temporal profile of the laser pulse is Gaussian with a pulse duration of 35 fs as the full width at half maximum of the intensity (FWHM). The time when the laser peak reaches the target is set as *t* = 0 fs. We use 500 cells per µm with 200 micro-particles per cell. The time step in the simulations is 2.7×10^-3^ fs. Open boundary conditions are used for both fields and particles.

The charge state distributions at *t* = 17 fs are plotted for both simulations in Fig. 3 (a). By comparing the results, we conclude that the collisional ionization appreciably alters the charge state distribution in the entire target. Specifically, we find that the number of ions with Z ≥ 22 increases as Cu^+20^ and Cu^+21^ ions get ionized to higher charge states, with most of the contribution coming from Cu^+20^ ions. Due to the collisional ionization to higher charge states from Cu^+21^, the quasi-monoenergetic component of Cu^+21^ (37 MeV) becomes much less pronounced [Fig. 4(a)]. It is worth pointing out that the histogram in Fig. 3 (a) represents all ions regardless of their energy.

The primary focus of this work is on high-energy high-Z ions, so we now examine the energetic part of the ion spectra in more detail. We find that the 1D simulations with and without the collisional ionization reproduce the key feature of the study, which is the presence of high-energy peaks in the spectra of high-Z ions (see Fig. 4(b) of this supplementary material and Fig. 5 of the main text). In 1D, the peaks correspond to energies that exceed 100 MeV for ions with Z ≥ 22. Figures 3 (b) and (c) show the charge state distributions for ions with energies above 100 MeV and 150 MeV, which effectively captures the peaks we are interested in. These figures clearly indicate that the collisional ionization does not affect the numbers and the peak structures of energetic and highly charged ions. So it is negligible for the ionization of these ions.

The difference between the characteristic time scales of the ion expansion and the collisional ionization is the primary cause for the apparent difference between the histograms in Fig. 3(a) and Fig. 3 (b) and (c). Once the main pulse reaches the surface of the target, a strong electrostatic sheath field is generated at the rear surface. Those target ions that are exposed to this field become ionized to high charge states while also being accelerated to energies exceeding 100 MeV. The local density of the accelerated ions rapidly decreases because these ions expand into the vacuum region [Fig. 6(a) in the main text]. The high-energy ions shown in Figs. 3(b) and 3(c) are ionized to charge states above Z = 21 primarily by the strong electric field that accelerates them. The collisional ionization remains unimportant as the density in the corresponding region continues to drop. In contrast to that, the ions with lower charge states of Z = 20 and Z = 21 have lower energies and stay in or close to the target bulk where the electric field is relatively weak and the plasma density is much higher than that at the location of high-energy ion bunches. Thus, the frequency of the collisional ionization that depends on the local density remains high enough over a much longer time period, which allows for significant ionization of Cu^+20^ to take place in high density regions. However, these ions never become energetic because the electric field drops on a time scale determined by the laser pulse duration and this time scale is typically shorter for our parameters than the ionization time.

We therefore conclude that the results presented in the main text for the high-Z, high-energy ion bunches should be relatively unaffected by the collisional ionization. However, the 2D simulation must include the collisional ionization process if the focus is on the low-energy part of the ion spectrum or on low-Z charge states. Such a simulation would be necessarily much more computationally intensive that the simulations we have performed. These arguments form the basis for our cost-benefit analysis that led us to conclude that including only the field ionization is appropriate for the problem of interest.


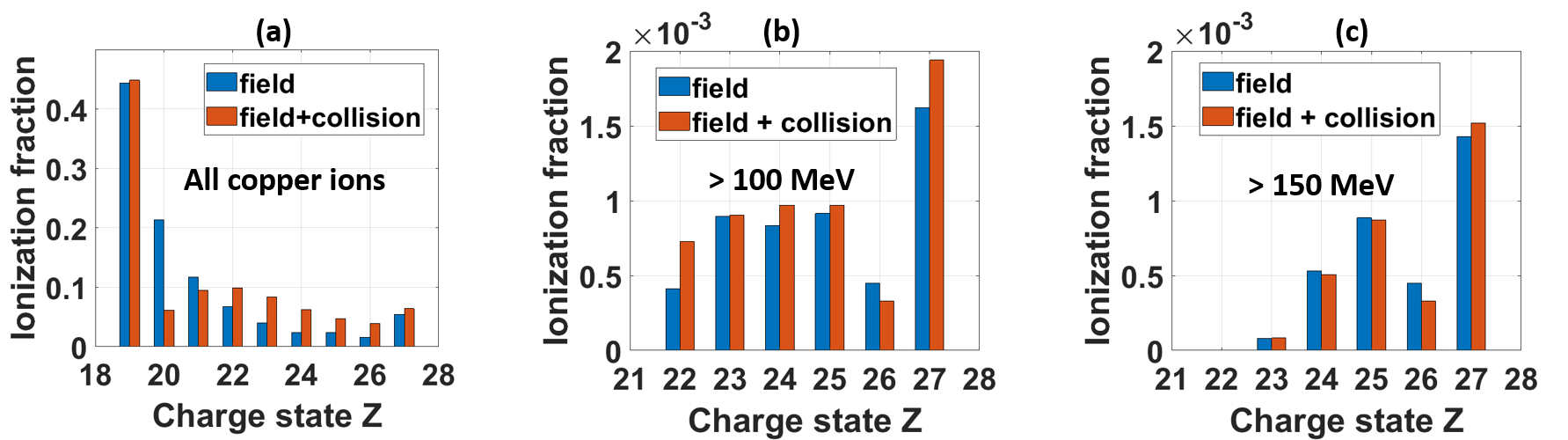


*Figure 3. The numbers of the L-shell ions normalized by the total ion number in two 1D PIC simulations with only field ionization (blue) and both field and collisional ionizations (red) at t=17 fs: (a) all the ions; (b) ions with energies above 100 MeV and (c) ions with energies above 150 MeV.*


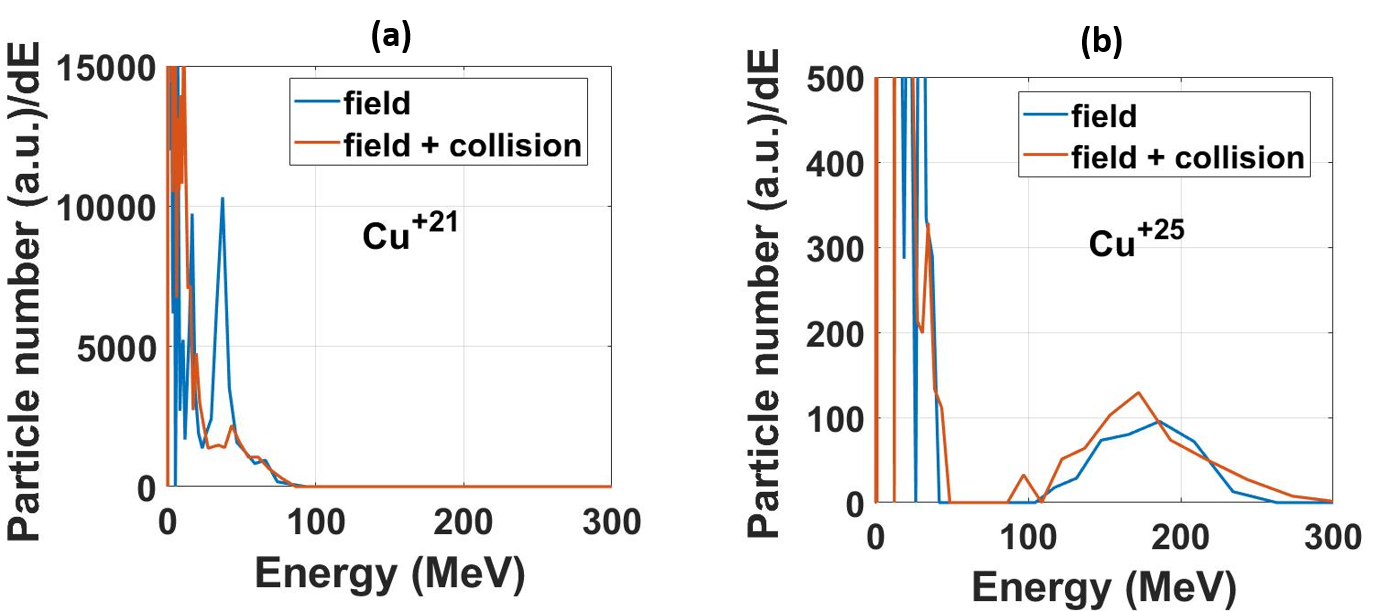


*Figure 4. The energy spectra of (a) Cu^+21^ and (b) Cu^+25^ at t = 17 fs in the simulations with only field ionization (blue) and with both field and collisional ionizations (orange).*

Photoionization

In the context of our problem, the photoionization becomes important during the main pulse and the subsequent ion acceleration process if it can produce L-shell ions from Cu^+19^ ions on this time scale, as this would lead to a disruption of the described ion bunch localization. The time that it takes to ionize a Cu^+19^ ion and turn it into a Cu^+20^ ion is

$t_{ph}=1/\int_{E_{*}}^{+\infty} n_{ph}f_{ph}(E)c\sigma(E)dE$ (1)

where $n_{ph}$ is the photon density, $E_{*}$ is the threshold ionization energy from Cu^+19^ to Cu^+20^, $f_{ph}\left( E \right)$ is the normalized photon energy spectrum [$\int_{0}^{+\infty} f_{ph}\left( E \right)dE=1$], c is the light speed and $\sigma(E)$ is the the cross section of the photoionization from Cu^+19^ to Cu^+20^ for a photon with energy *E* [12].

Our simulations provide no information about the photons, so we use the information available in the literature to estimate $f_{ph}\left( E \right)$ and $n_{ph}$ for our laser parameters. It has been previously shown that the photons are well-described using a thermal distribution with an effective temperature *T_ph_*:

$f_{ph}\left( E \right)=\frac{1}{T_{ph}}exp(-E/T_{ph}$), (2)

where the distribution is normalized as described earlier. Note that there is typically an additional high-energy tail that has a much higher temperature. We neglect this part of the distribution because these photons contribute very little to the ionization of Cu^+19^ due to their high energy. The photon density can be roughly estimated as

$n_{ph}\approx E_{total}\alpha/(T_{ph}$c$\tau S)$ (3)

where *α* is the energy conversion rate from the laser to photons, and *E_total_*, $\tau$ and *S* are the incident laser energy, duration, and the area of the focal spot.

Equation (1) can now be simplified by also taking into account that $\sigma(E)\propto E^{-3}$ [12] in the keV range. We set $\sigma(E)=\sigma_{*}\left( E/{E_{*}} \right)^{-3}$, where $\sigma_{*}=\sigma\left( E_{*} \right)=5\times{10}^{-20}{cm}^{2}$ is the photoionization cross section of Cu^+19^ at the threshold energy $E_{*}$=1.7 keV. We assume that *T_ph_* >>$E_{*}$, which then allows us to approximately evaluate the integral in the denominator of equation (1) and find that

$t_{ph}\approx\frac{2T_{ph}}{\sigma_{*}cn_{ph}E_{*}}=\frac{{2T}_{ph}^{2}}{{\alpha E}_{*}E_{total}}\frac{\tau S}{\sigma_{*}}$. (5)

We estimate the key parameters α and *T_ph_* by assuming that the bremsstrahlung process is the main process for the photon emission in our regime of interest. The conversion efficiency for the bremsstrahlung is sensitive to the material thickness and to the atomic type. We use the results of two previous studies [13, 14] where the conversion efficiency was estimated as *α* ~ 10^-5 ~ -4^  for similar laser parameters. In these studies, a higher-Z material was used and the target was much thicker than 20 nm, so they provide an upper estimate for the conversion rate. We choose a maximum value of *α* = 10^-4^ to obtain a lower limit for the photoionization time.

We estimate *T_ph_* based on our own simulation results for the electron temperature. The photon temperature of the bremsstrahlung produced photons is usually comparable to the temperature of the electron population that generates these photons [15–17]. In our case, the electron distribution has two distinct components: one is the high energy component with *T_eh_*=3~5MeV and the other is the cold energy component with *T_ec_*=300~400keV. Since the ionization cross section decrease as ~E^-3^, the contribution by the hot component is relatively insignificant. Therefore, we only consider the cold electron temperature component for estimating the photoionization rate and set *T_ph_*= 400 keV.

In order to estimate the ionization time, we assume that the laser pulse in our simulations represents a 3D laser pulse with *T_otal_* ~6 J, $\tau$= 35 fs and *S* =1.3×10^-7^ cm^2^, where the area of the focal spot is calculated based on the transverse beam size provided in the main text. We find from Eq. (5) that *t_ph_* ~ 4.4 ns for *T_ph_* = 400 keV and *α* = 10^-4^. This time exceeds the laser pulse duration by four orders of magnitude, so we conclude that the photoionization should be negligible in our problem. Even if the photon temperature is significantly reduced, *T_ph_* = 4 keV, while the conversion rate is increased to *α* = 0.001, the ionization time, *t_ph_* ~ 44 fs, still remains longer than the laser pulse duration.

**References**

1. Dorrer, C., Begishev, I. a, Okishev, a V & Zuegel, J. D. High-contrast optical-parametric amplifier as a front end of high-power laser systems. *Optics letters* **32,** 2143 (2007).

2. Jullien, A. *et al.* 10^-10^ Temporal Contrast for Femtosecond Ultraintense Lasers By Cross-Polarized Wave Generation. *Optics Letters* **30,** 920 (2005).

3. Kalashnikov, M. P., Risse, E., Schönnagel, H. & Sandner, W. Double chirped-pulse-amplification laser: a way to clean pulses temporally. *Opt. Lett.* **30,** 923 (2005).

4. Hillier, D. *et al.* Ultrahigh contrast from a frequency-doubled chirped-pulse-amplification beamline. *Appl. Opt.* **52,** 4258 (2013).

5. Itatani, J., Faure, J., Nantel, M., Mourou, G. & Watanabe, S. Suppression of the amplified spontaneous emission in chirped-pulse-amplification lasers by clean high-energy seed-pulse injection. *Optics Communications* **148,** 70 (1998).

6. Thaury, C. *et al.* Plasma mirrors for ultrahigh-intensity optics. *Nat. Phys.* **3,** 424–429 (2007).

7. MacFarlane, J. J., Golovkin, I. E. & Woodruff, P. R. HELIOS-CR - A 1-D radiation-magnetohydrodynamics code with inline atomic kinetics modeling. *J. Quant. Spectrosc. Radiat. Transf.* **99,** 381–397 (2006).

8. Torrisi, L. *et al.* Comparison of nanosecond laser ablation at 1064 and 308 nm wavelength. *Appl. Surf. Sci.* **210,** 262–273 (2003).

9. Ilyas, B., Dogar, A. H., Ullah, S. & Qayyum, A. Laser fluence effects on ion emission from a laser-generated Cu plasma. *J. Phys. D. Appl. Phys.* **44,** 295202–295208 (2011).

10. Kawahito, D. & Kishimoto, Y. Multi-phase ionization dynamics of carbon thin film irradiated by high power short pulse laser. *Phys. Plasmas* **24,** 103105 (2017).

11. Kim, Y.-K. & Rudd, M. E. Binary-encounter-dipole model for electron-impact ionization. *PHYSICAL REVIEW A* **50,** 3954 (1994)

12. Verner, D. A., Ferland, G. J., Korista, K. T. & Yakovlev, D. G. Atomic Data for Astrophysics. II. New Analytic FITS for Photoionization Cross Sections of Atoms and Ions. *Astrophys. J.* **465,** 487 (1996).

13. Sentoku, Y., Mima, K., Taguchi, T., Miyamoto, S. & Kishimoto, Y. Particle simulation on x-ray emissions from ultra-intense laser produced plasmas. *Phys. Plasmas* **5,** 4366–4372 (1998).

14. Wan, F., Lv, C., Jia, M., Sang, H. & Xie, B. Photon emission by bremsstrahlung and nonlinear Compton scattering in the interaction of ultraintense laser with plasmas. *Eur. Phys. J. D* **71,** 236 (2017).

15. Sawada, H. *et al.* Characterization of intense laser-produced fast electrons using hard x-rays via bremsstrahlung. *J. Phys. B At. Mol. Opt. Phys.* **48,** 224008 (2015).

16. Schwoerer, H. *et al.* MeV X rays and photoneutrons from femtosecond laser-produced plasmas. *Phys. Rev. Lett.* **86,** 2317–2320 (2001).

17. Zulick, C. *et al.* High resolution bremsstrahlung and fast electron characterization in ultrafast intense laser-solid interactions. *New J. Phys.* **15,** 123038 (2013).
